# Power Analysis and Effect Size in Mixed Effects Models: A Tutorial

**DOI:** 10.5334/joc.10

**Published:** 2018-01-12

**Authors:** Marc Brysbaert, Michaël Stevens

**Affiliations:** 1 Department of Experimental Psychology, Ghent University, Henri Dunantlaan 2, B-9000 Gent, BE; 2 Ghent University, BE

**Keywords:** power analysis, effect size, mixed effects models, random factors, F1 analysis, F2 analysis

## Abstract

In psychology, attempts to replicate published findings are less successful than expected. For properly powered studies replication rate should be around 80%, whereas in practice less than 40% of the studies selected from different areas of psychology can be replicated. Researchers in cognitive psychology are hindered in estimating the power of their studies, because the designs they use present a sample of stimulus materials to a sample of participants, a situation not covered by most power formulas. To remedy the situation, we review the literature related to the topic and introduce recent software packages, which we apply to the data of two masked priming studies with high power. We checked how we could estimate the power of each study and how much they could be reduced to remain powerful enough. On the basis of this analysis, we recommend that a properly powered reaction time experiment with repeated measures has at least 1,600 word observations per condition (e.g., 40 participants, 40 stimuli). This is considerably more than current practice. We also show that researchers must include the number of observations in meta-analyses because the effect sizes currently reported depend on the number of stimuli presented to the participants. Our analyses can easily be applied to new datasets gathered.

A revolution is taking place in the statistical analysis of psychological studies. Whereas before, analyses were limited to designs with a single random variable (either participants in so-called F1 analyses, or stimuli in so-called F2 analyses), mixed effects models currently allow researchers to take into account both participants and stimuli as random variables (Baayen, Davidson, & Bates, 2008; Judd, Westfall, & Kenny, 2017).

Because the new analysis techniques are still being discovered, there is a need for papers explaining their use. The present paper examines the issues of power and effect size.

## Many experiments in psychology are underpowered

Ever since Cohen’s (1962) classic study on statistical power, it has been known that many psychology studies contain too few observations to properly investigate the effects under examination. A good experiment has 80% a priori chance of finding the effect, given that the effect is present at the population level. The 80% is a compromise between certainty about the effect and the investments needed to further increase the power. Even so, Cohen (1962) showed that many experiments published in the established psychology journals have a power considerably below 80% (often as low as 30–40%). There is little evidence that the situation nowadays is much better than in the 1960s (Button, Ioannidis, Mokrysz, Nosek, Flint, Robinson, & Munafò, 2013; Dumas-Mallet, Button, Boraud, Gonon, & Munafo, 2017; Smaldino, & McElreath, 2016; Vankov, Bowers, & Munafò, 2014).

The necessary number of observations depends on the difference between the conditions. When the difference is large (e.g., the difference in height between children of 3 years old and adolescents of 18 years old), one only needs a few observations per group. When the difference is small (e.g., the difference in height between adolescents of 16 years and adolescents of 18 years), one requires many more observations to come to a conclusion.

The difference between conditions is usually expressed as a standardized effect size, an effect size independent of the measurement unit. Two popular measures are Cohen’s d and η^2^ (pronounced eta-squared). Cohen’s d expresses the difference between two means relative to their standard deviation [so, d = (mean 1–mean 2)/(the average standard deviation of the two groups)]. Eta squared indicates how much of the total variance in the data is explained by the difference between the means. You can go from d to η^2^ with the equation: \eta^{2} = {{d^{2}} \over {d^{2} + 4}}, and vice versa with the equation: d = \sqrt {4\eta^{2} \over {1-\eta^{2}}}.

A typical effect size in psychology is d = .4 (η^2^ = .04; Kuhberger, Fritz, & Scherndl, 2014; Open Science Collaboration, 2015). This is small, requiring many observations. For comparison purposes, the effect size of the difference in height between male and female adults is d = 2.3.

Several applets are available to calculate the number of observations needed for 80% power when the effect size is d = .4. According to Lenth’s website (https://homepage.stat.uiowa.edu/~rlenth/Power/) you need two groups of 99 persons to find such an effect in a between-subjects design, and 51 participants in a repeated measures design. If you want to play safe and assume an effect size of d = .3, you require 175 participants per condition in a between-subjects design, and 89 participants in a repeated measures design.^1^ These are numbers rarely seen in psychological research, meaning that many studies are underpowered. As a result, the Open Science Collaboration (2015) could replicate only 36 of the 100 studies selected from different areas of psychology, rather than the 80% expected. An even lower rate was reported by Marsman, Schönbrodt, Morey, Yao, Gelman, and Wagenmakers (2017) for a series of registered replications in social psychology. At the same time, it is becoming clear that the definition of replication success may be less straightforward than assumed at first (Amrhein, Korner-Nievergelt, & Roth, 2017; Etz & Vandekerckhove, 2016).

The consequences of underpowered studies are becoming increasingly clear (Loken & Gelman, 2017). First, p-values show wide sample-to-sample variability, particularly when they are based on studies with small sample sizes (Cumming, 2014; Halsey, Curran-Everett, Vowler, & Drummond, 2015). Second, there is a file drawer problem (Fanelli, 2011; Franco, Malhotra, & Simonovits, 2014). Results with p < .05 are more likely to be published. Because such p-values require large observed effect sizes in small designs, the effects sizes reported in the literature tend to be inflated, also those from studies that can be replicated (Kuhberger et al., 2014; Open Science Collaboration, 2015; Vasishth & Gelman, 2017). Third, the file drawer problem combined with low power not only results in published papers with effects that do not exist at the population level, but also leads to an unacceptably high number of publications reporting and interpreting effects opposite to the one present at the population level (Gelman & Carlin, 2014). Fourth, researchers not only look at the effects they were interested in at the outset of their study, but they tend to interpret all statistically significant effects and sometimes even rephrase their hypotheses on the basis of the data obtained (a phenomenon known as harking – hypothesizing after the results are known; Kerr, 1998). Finally, the above problems are exacerbated by researchers massaging their data to get promising trends beyond the p < .05 ‘significance’ level. As a result, chances of publishing type I errors are considerably higher than the 5% expected when one uses the p < .05 criterion (Francis, 2012; Leggett, Thomas, Loetscher, & Nicholls, 2013; Simmons, Nelson, & Simonsohn, 2011). Only an environment in which underpowered studies are not rewarded will lead to a decrease of the problem of such studies (Smaldino, & McElreath, 2016).

## What about experiments with multiple observations per participant per condition?

Many researchers in cognitive psychology have wondered to what extent the power studies reported in the literature apply to them. After all, they regularly replicate effects with some 20 participants in repeated measures designs, meaning that they must be working with effect sizes of d > .66. This raises the question whether the effect sizes in cognitive psychology experiments are so much bigger than those observed in applied settings.

One possibility is that researchers in cognitive psychology usually have multiple observations per participant per condition. Take, for instance, a researcher investigating the frequency effect in word recognition. The word frequency effect says that words occurring often in the language (high frequency words) will be processed faster than words occurring rarely in the language (low-frequency words). A researcher investigating the effect is unlikely to present but one high-frequency and one low-frequency word to each participant. Instead, they will present some 40 high-frequency words and some 40 low-frequency words, and take the average reaction times for each participant as the dependent variable. Could this be the reason why effect sizes are bigger in cognitive psychology than in other areas of psychology and, if so, how does the power of a study relate to the number of participants tested and the number of stimuli administered? These are the questions addressed in the sections below.

## The first database used: Adelman et al. (2014)

In our discussion we will work with two overpowered datasets, because they help us to understand what happens when studies have less power. The first study was published by Adelman, Johnson, McCormick, McKague, Kinoshita, Bowers, Perry, Lupker, Forster, Cortese, Scaltritti, Aschenbrenner, Coane, White, Yap, Davis, Kim, & Davis (2014). In total they examined 1020 participants who responded to 420 words in a lexical decision task (is the stimulus a word: yes/no). There were also 420 trials with nonwords per participant, but we are not interested in those.

Adelman et al. (2014) studied the effects of orthographic priming. The target words were presented in uppercase letters and were preceded by lowercase primes that varied from completely identical to the target word (design-DESIGN) to completely different (voctal-DESIGN). An orthographic priming effect is observed when reaction times to target words are faster when they are preceded by related primes (design-DESIGN) than by unrelated primes (voctal-DESIGN). There were 28 different types of primes in the Adelman et al. study with varying degrees of overlap (such as degn-DESIGN or idgens-DESIGN). As small differences were expected between several of these prime types, Adelman et al. (2014) wanted a study with confidence intervals of some 2 ms around the obtained priming effects. Hence, the large number of participants and stimuli. Primes were presented for 50 ms, so that they were next to invisible to the participants (masked priming).

**Figure 1:**
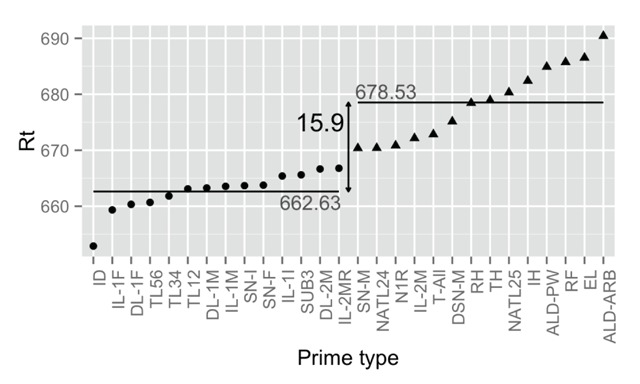
Construction of the two prime types from the data of the Adelman et al. (2014) priming megastudy. Prime types varied from an identity prime (extreme left) to an all letter different prime (extreme right).

**Figure 2:**
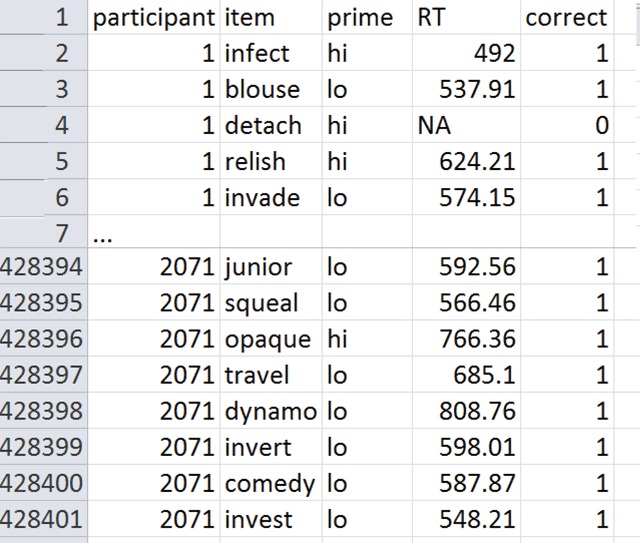
Snapshot of the Adelman et al. (2014) database used. Participant is the rank number of the participant tested (not all participants who started the study provided useful results); item = the target word responded to; prime = highly or lowly related to the target; RT is the reaction time to the target in the lexical decision task; correct = whether or not the answer was correct.

To simplify the dataset, we ordered the prime types according to average RT and split them in two groups: highly related vs. lowly related, as shown in Figure 1. This resulted in two conditions with 214,200 trials per condition and a priming effect of 16 ms. As is general practice, RTs of error trials and outliers were excluded (outliers were detected using an adjusted boxplot for skewed distributions, Hubert & Vandervieren, 2008). Together these criteria resulted in a loss of 12.1% of the data and 376,476 remaining observations. The data and the analyses we ran are available as supplementary files. Figure 2 gives a snapshot of the stimulus file used.

### Traditional F1 and F2 analyis

Up to recently, the data of Adelman et al. (2014) were analyzed with two separate analyses: one across participants (F1) and one across items (F2). In the F1 analysis, per participant the average RT was calculated for all words preceded by a related prime and for all words preceded by an unrelated prime. Then, the average values per participant were entered into an analysis of variance with one repeated measure (two levels) or a paired t-test. In the F2 analysis, means were calculated for every item, which were subsequently entered into an analysis of variance. Table 1 shows the outcome of these two analyses.

**Table 1:** Outcome of a traditional F1 and F2 analysis of the Adelman et al. (2014) dataset.

F1 analysis
N_participants_ = 1020, RT_related condition_ = 660.0, RT_unrelated condition_ = 674.9 F1(1,1019) = 766.1, MSe = 146.98, p < .01 d = .87
**F2 analysis**
N_items_ = 420, RT_related condition_ = 661.4, RT_unrelated condition_ = 677.6 F2(1,419) = 649.3, MSe = 84.87, p < .01 d = 1.24

Table 1 illustrates the complications faced by researchers when reporting the outcome of F1 and F2 analyses:

- Because of the missing values (errors, outliers): the means of the F1 and the F2 analysis are not the same.
- There is no straightforward and well-established way to combine F1 and F2 into a single measure indicating the significance of the effect. However, given that the effect is (highly) significant both in the F1 and F2 analysis, we can assume that it generalizes across participants (F1) and across stimuli (F2).
- The effect sizes differ between F1 and F2. This creates difficulties for meta-analysis (which one to choose?).

The traditional F1 and F2 analyses suggest that the effect size investigated by Adelman et al. (2014) is large (d > .8). Hence, the study is seriously overpowered for our purpose (remember that Adelman et al. wanted to compare much smaller differences in priming effects). The simplified case allows us, however, to ask how many observations are needed for a properly powered experiment with two conditions.

### A linear mixed effects analysis

Because the design of Adelman et al. (2014) contains two random variables (participants and items), it would be better if a single analysis could take them both into account. Such analysis has become available (Baayen, et al., 2008; Judd, Westfall, & Kenny, 2017). We will make use of the lme4 package developed for R by Bates, Mächler, Bolker, and Walker (2015).

The code is remarkably simple:

library(lme4)
fit <- lmer(RT ~ prime + (prime|item) + (prime|participant), data = adelman)
summary(fit)

In this analysis, there is one fixed effect (the effect of prime) and four random effects:

1. The intercept per participant (capturing the fact that some participants are faster than others).
2. The intercept per item (capturing the fact that some items are easier than others).
3. The slope per participant (capturing the possibility that the priming effect is not the same for all participants).
4. The slope per item (capturing the possibility that the priming effect is not the same for all items).

The outcome of the mixed effects analysis is shown in Table 2. It tells us that the estimated difference between the related and the unrelated prime condition is 16.0 ms and that it is significant (t = 28.78, which equals to F = t² = 828). The test statistic again confirms that the study was overpowered.

**Table 2:** Outcome of the lmer analysis (Bates et al., 2015) of the Adelman et al. (2014) dataset.

Random effects			
Groups Name	Variance	Std.Dev.	Corr

participant (Intercept)	10032.34	100.162	
prime/participant	27.89	5.282	–0.40
item (Intercept)	1900.12	43.590	
prime/item	19.88	4.458	0.53
Residual	22128.15	148.755	
Number of obs: 376476, groups: participant, 1020; item, 420			
**Fixed effects**			
	**Estimate**	**Std. Error**	**t value**

(Intercept)	662.582	3.805	174.13
Prime (lo vs. hi)	16.029	0.557	28.78
Estimated RTs are: related = 662.6 ms, unrelated = 678.6 ms			
**Correlation of Fixed Effects**			
	**(Intr)**		
		
Prime	–0.036		

## Effect size and power analysis: Westfall, Judd, and Kenny (2014)

Westfall et al. (2014) published a theoretical analysis of mixed effects models and a website allowing researchers to run power analysis for simple designs with one fixed effect and two random factors.

First, Westfall et al. (2014) showed how you can calculate the effect size (measured as d) for a design with random participants and random items. The equation is as follows:

\begin{array}{l}
d = \,\,\frac{{difference\, between\, the\, means}}{{\sqrt {varintercep{t_{part}} + varintercep{t_{item}} + varslop{e_{part}} + varslop{e_{item}} + va{r_{residual}}}}}\\
d = \,\,\frac{{16.029}}{{\sqrt {10032 + 1900 + 27.9 + 19.9 + 22129}}}\\
d = \,\,\,.0868
\end{array}

Attentive readers have noticed the vast difference between the d-value calculated for the mixed effects analysis and the d-values calculated for the F1 and F2 analyses (Table 1). This points us to a first, important insight: *The d-value of an F1 analysis depends on the number of items per condition, and the d-value of the F2 analysis depends on the number of participants in the study*.

Table 3 shows how the effect size in the F1 and F2 analysis depends on the number of observations over which the data are averaged. In hindsight, this is stating the obvious because averaging reduces the standard errors, but to our knowledge no-one has made the link before. In particular, it means that the effect sizes of experimental data published in meta-analyses are conditional on the number of items used in the various studies. It also explains why the effect size was larger in the item analysis (averaged over 1020 participants) than in the participant analysis (averaged over 420 items). As it happens, when the numbers are equalized, the effect sizes in the F1 and F2 analyses in the Adelman et al. database largely converge (because the variability across participants in the dataset is very similar to the variability across items).

**Table 3:** Illustration of how the effect size in the F1 analysis depends on the number of stimuli over which the participant means are averaged, and how the effect size in the F2 analysis depends on the number of participants over which the item means are averaged. Values obtained by drawing random samples of N from the Adelman et al. (2014) database.

Effect size of the F1 analysis with all participants when the number of stimuli is limited to:	
Nitems = 20	d = .19
Nitems = 40	d = .28
Nitems = 80	d = .39
Nitems = 160	d = .55
Nitems = 320	d = .77
Nitems = 420	d = .87
Effect size of the F2 analysis with all items included when the number of participants is limited to:	
Nparts = 20	d = .18
Nparts = 40	d = .26
Nparts = 80	d = .37
Nparts = 160	d = .52
Nparts = 320	d = .74
Nparts = 640	d = 1.02
Nparts = 1020	d = 1.24

The value d = .0868 in the mixed effects analysis illustrates that a difference of 16 ms is very small when compared to RTs that can vary from 250 ms to 1500 ms. Indeed, the standard deviation calculated across all 376,476 valid observations from the Adelman et al. (2014) dataset is 182.4. Given such a level of noise, a difference of 16 ms translates to an estimate of d ≈ 16/182.4 ≈ .087. The higher d-values in the F1 and F2 analyses are obtained by averaging across observations, which reduces the variance in the data on which the F1 and F2 analyses are based.

## Calculating the number of participants for a properly powered experiment with participants and items as random factors according to Westfall et al. (2014)

Westfall et al. (2014) not only published a theoretical analysis of the mixed effects model, they also made a website (https://jakewestfall.shinyapps.io/two_factor_power/) which allows researchers to calculate the power of an experiment and the number of items/participants required for a well-powered experiment.

What one needs for the website, is an estimate of the effect size and six so-called Variance Partitioning Coefficients (VPCs). The latter sound scarier than they are. You obtain them by calculating the proportion of each random variance component relative to their sum. So, the various VPCs are:

\begin{array}{c}
VP{C_{interceptpart}} = \,\,\,\frac{{10032}}{{10032 + 1900 + 27.9 + 19.9 + 22129}}\,\,\, = .29412\\
VP{C_{interceptitem}} = \,\,\,\frac{{1900}}{{10032 + 1900 + 27.9 + 19.9 + 22129}}\,\,\, = .05570\\
VP{C_{slopepart}} = \,\,\,\frac{{27.9}}{{10032 + 1900 + 27.9 + 19.9 + 22129}}\,\,\, = .00082\\
VP{C_{slopeitem}} = \,\,\,\frac{{19.9}}{{10032 + 1900 + 27.9 + 19.9 + 22129}}\,\,\, = .00058\\
VP{C_{residual}} = \,\,\,\frac{{22129}}{{10032 + 1900 + 27.9 + 19.9 + 22129}}\,\,\, = .64878
\end{array}

The five components must add to 1.0. The sixth component (VPC_participant*item_) cannot be estimated for a counterbalanced design (with one observation per participant per item) and can be set to 0.

**Figure 3:**
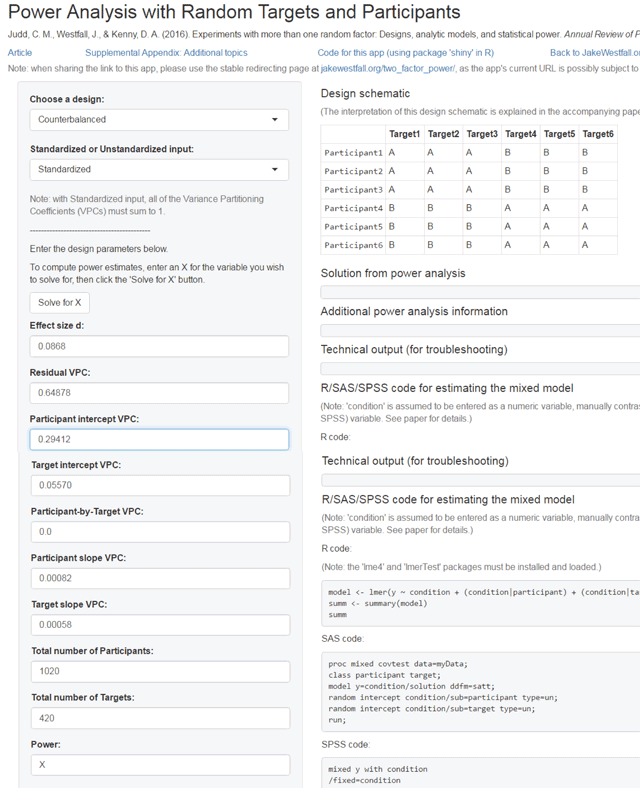
Input in the Westfall et al. (2014) website to calculate power of a simple design with random effects of participants and targets (items). Data based on the lmer analysis of the Adelman et al. (2014) dataset.

If we run the analysis for the complete design (Figure 3), we get a power ≈ 1.00, meaning we will almost always find a significant p < .05 difference between the related and the unrelated condition. More interesting is to see how many items we would need if we only had 40 participants. The calculations come to an estimate of 83 stimuli, which would translate to some 96 (48 per condition) if we take the 12% data loss into account. Similarly, if we have only 50 items (25 per condition), we need 65 + 12% ≈ 74 participants.

The analysis of Westfall et al. (2014) further indicates that it is meaningless to set up designs with too few participants or too few stimuli, because the number of the other variable rapidly increases and even goes to infinity for small numbers of participants or stimuli.

## Calculating the number of participants and items for a properly powered experiment on the basis of simulation

Westfall et al.’s (2014) theoretical approach has two limitations:

1. It only works for designs with one fixed effect with two levels.
2. The estimates differ considerably when different values are entered. This is a problem with RTs, because some estimates are rather small and unstable due to the large residual component.

An alternative is to work with simulations. Simulations can be run for all possible designs and are more robust. Simulations can either be written by the user (if you have the skills) as shown in Stevens and Brysbaert (2016), or we can make use of preprogrammed software packages. A particularly interesting R package is simR (Green & MacLeod, 2016; Green, MacLeod, & Alday, 2016).^2^

To estimate the power of the Adelman et al. (2014) design, it suffices to enter the following commands:

library(simr)
fit <- lmer(RT ~ prime + (prime|item) + (prime|participant), data = adelman)
power <- powerSim(fit,nsim = 200)
power

This will estimate the power on the basis of 200 random samples (more are possible if your computer is fast enough). It will again tell you that the Adelman et al. study is overpowered (i.e. next to all 200 simulations will return a significant effect).

To find out which combinations of numbers of participants and numbers of participants are good, one can sample various sizes from the original database and see how often the program returns a significant effect.

The following program takes 100 random samples of 40 participants and 40 items and estimates the power based on 20 simulations each.

#make sure you have all the names of the items and the participants in the database
itm <- unique(adelman$item)
part <- unique(adelman$participant)
 
#calculate the power of 100 samples of 40 participants and 40 items each
pow = list()
for (i in 1:100) {
   print(i)
   selectionpart <- sample(part$participant,40)
   selectionitem <- sample(itm$item,40)
   adelman2 <- adelman[which(adelman$participant %in% selectionpart & adelman$item %in% selectionitem), ]
   fit <- lmer(RT ~ prime + (prime|item) + (prime|participant), data = adelman2)
   power <- powerSim(fit,nsim = 20)
   pow[i] <- power[1]
}
p <- unlist(pow)
p = p*5
mean(p)
hist(p)

This analysis shows that with 40 participants and 40 items, we have an average power of .41 (this analysis takes into account the 12% data loss due to errors and outliers). Table 4 shows the power for various numbers of participants and items. It tells us that the estimates of Westfall et al. (2014) are too optimistic. For the Adelman et al. (2014) database, one requires some 6,000 observations for a properly powered experiment (e.g., 60 participants and 100 stimuli, or 80 participants and 80 stimuli, or 100 participants and 60 stimuli).

**Table 4:** Power in the Adelman et al. (2014) study when estimated on the basis of simulation. Numbers not given are all > 80. This table shows that the 16 ms effect in the study could reliably be detected with some 6,000 observations (60 participants, 100 stimuli; 80 participants, 80 stimuli; 100 participants, 60 stimuli). The standard errors of the estimates are about 2.5 (i.e. the confidence interval of the power estimate of 17% in the 20 participants 20 items condition goes from 12% to 22%).

Nparts							
Nitems	20	40	60	80	100	120	1020

**20**	17	25	32	42	46	51	99.8
**40**	21	41	51	69	72	76	
**60**	28	52	64	76	88		
**80**	37	62	77	83			
**100**	41	70	84				
**120**	47	74					
**420**	86						

Table 4 also shows us that with 420 stimuli power is good enough for 20 participants already to observe a priming effect of 16 ms. With 1020 participants, 20 stimuli are more than enough as well.

## Changing the dependent variable to inverse RTs

A concern about RTs is that their distribution is positively skewed. So, they violate the assumption of normally distributed variables underlying analyses of variance. Two transformations are possible: either taking the logarithm of RT, or taking the inverse RT (which results in units of information processed per ms). In particular the latter is becoming increasingly used for mixed effects models. In our analyses we also saw that it resulted in better performance than log(RT).

Table 5 gives the various calculations for inverse RTs, defined as invRT = {{-1000} \over {RT}}. The nominator was set to –1000 in order not to have too small values and to make sure that the related condition with the lowest RT also had the lowest invRT.

**Table 5:** Outcome of analyses based on invRT for the Adelman et al. (2014) database, showing that the analysis of invRT is more powerful than the analysis of RT. Numbers not shown in the power analysis table are >80.

F1 analysis														
N_participants_ = 1020, invRT_related condition_ = –1.61 (RT = 1000/1.61 = 621 ms), invRTunrelated condition = –1.57 (RT = 1000/1.57 = 637 ms)														
F1(1,1019) = 1153, MSe = 0.0007, p < .01														
d = 1.06														
**F2 analysis**														
N_items_ = 420, invRT_related condition_ = –1.61 (RT = 1000/1.61 = 621 ms), invRT_unrelated condition_ = –1.57 (RT = 1000/1.57 = 637 ms)														
F2(1,419) = 1000, MSe = 0.0004, p < .01														
d = 1.54														
**lme analysis**														
**Random effects**														
	**Groups Name**	**Variance**	**VPC**	**Corr**										

	participant (Intercept)	0.0490519	0.368											
	prime/participant	0.0004761	0.004	–0.78										
	item (Intercept)	0.0091417	0.068											
	prime/item	0.0001626	0.001	0.10										
	Residual	0.0746261	0.559											
	Number of obs: 376476, groups: participant, 1020; item, 420													
**Fixed effects**														
		**Estimate**	**Std. Error**	**t value**										

	(Intercept)	–1.604953	0.008382	–191.48										
	prime	0.041329	0.001285	32.17										
	Estimated RTs are: related= –1000/–1.605 = 623 ms, unrelated = –1000/(–1.605+.041) = 639 ms													
**Correlation of Fixed Effects**														
		**(Intr)**												
	
	Prime	–0.354												
Effect size Westfall et al.: d = \,\,\,\frac{{.041}}{{\sqrt {.04905 + .00048 + .00914 + .00016 + .07463}}}\,\,\, = .112														
**Power analysis on the basis of simulation**														
	**Nparts**														
	**Nitems**	**20**	**40**	**60**	**80**	**100**	**120**	**1020**						
	
	**20**	21	39	45	54	61	71	100						
	**40**	37	59	76	86									
	**60**	47	74	88										
	**80**	54	84											
	**100**	67												
	**120**	68												
	**420**	97												

A comparison of the analyses with invRT and those with RT indicates that the former is more powerful than the latter. It results in less noise (residual variance) and higher estimates of the fixed effect(s) and random effects. The power analysis suggests that with invRT as dependent variable, one can properly test the 16 ms effect in the Adelman et al. study with some 3,200 observations (40 participants, 80 stimuli; 60 participants, 60 stimuli; 80 participants, 40 stimuli).

## A comparison dataset: Perea et al. (2015)

In the previous analyses we saw that the Adelman et al. (2014) dataset requires 3,200 observations to find a priming effect of 16 ms in a well-powered experiment. Some readers may feel hesitative about our use of this database, however. First, as illustrated in Figure 1 the two conditions we defined consisted of several subconditions with varying priming effects; this increases the noise in the priming effect. Second, the data were obtained from 14 different universities with quite large differences in average RTs (see Brysbaert, Lagrou, & Stevens, 2017, for a summary).

So, we might wonder how the data compare to those from a more “typical” experiment. For this we turn to a study published by Perea, Vergara-Martínez, and Gomez (2015). These authors also investigated masked orthographic priming (prime duration of 33 ms + 17 ms hash marks after the prime). They were particularly interested in repetition priming: the priming observed when a target is preceded by itself or by an unrelated prime. As shown in Figure 1, repetition priming is the strongest form of orthographic priming one can observe and regularly results in priming effects of over 30 ms.

**Figure 4:**
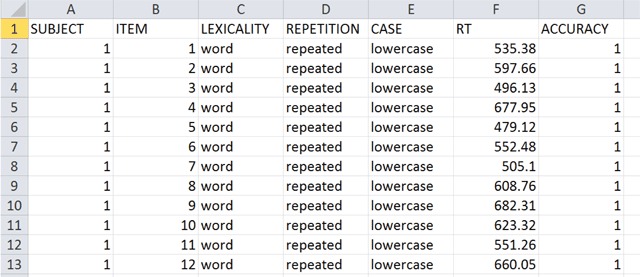
Top of the Perea et al. (2015) database.

Perea et al. (2015) wanted to know whether CaSe AlTeRnAtIoN reduces the priming effect. So, they had four conditions: uppercase targets preceded by lowercase related primes (regla – REGLA; the study was in Spanish), uppercase targets preceded by lowercase unrelated primes (civil – REGLA), uppercase targets preceded by alternating case related primes (rEgLa – REGLA), and uppercase targets preceded by alternating case unrelated primes (cIvIl – REGLA). There were 120 target words and 120 nonwords. The task was lexical decision and a total of 40 participants took part. Only the word trials are of interest to us. This resulted in a database of 120 × 40 = 4800 trials. Figure 4 gives a snapshot of the database.

Following the article of Perea et al. (2015), RTs to errors and RTs smaller than 250 ms and larger than 1500 ms were excluded, leading to a data loss of 6% (or 4512 remaining observations). Perea et al. (2015) found a main effect of repetition priming (as expected), no effect of case, and no interaction. To simplify matters, we deleted the nonsignificant Case variable and assumed there is only one fixed effect with two levels in the design (repeated vs. unrelated prime).^3^

As the invRT analysis was more powerful, only this one is given (Perea et al. also used this analysis in their article). Table 6 shows the results. Checking the outcome of the mixed effects analysis indicated that the random slopes per item did not add to the model. Therefore, this factor was dropped.^4^ The following lines show the three models: fit1 = the full model as before, fit2 = a model with no random effect of slope per item, fit3 = a model with no random effect of slope per participant.

fit1 <- lmer(invRT ~ REPETITION + (REPETITION|ITEM) + (REPETITION|SUBJECT), data = perea)
fit2 <- lmer(invRT ~ REPETITION + (1|ITEM) + (REPETITION|SUBJECT), data = perea)
fit3 <- lmer(invRT ~ REPETITION + (REPETITION|ITEM) + (1|SUBJECT), data = perea)
anova(fit1,fit2): chi sq(df = 2) = 2.3, n.s.
anova(fit1,fit3) : chi sq(df = 2) = 16.3, p < .01

**Table 6:** Outcome of analyses based on invRT for the Perea et al. (2015) database.

F1 analysis				
N_participants_ = 40, invRT_related condition_ = –1.756 (RT = 1000/1.756 = 569ms), invRT_unrelated condition_ = –1.647 (RT = 1000/1.647 = 607 ms)				
F1(1,39) = 77.81, MSe = 0.00306, p < .01				
d = 1.39				
**F2 analysis**				
N_items_ = 120, invRT_related condition_ = –1.757 (RT = 1000/1.757 = 569ms), invRT_unrelated condition_ = –1.645 (RT = 1000/1.645 = 608ms)				
F2(1,119) = 120.9, MSe = 0.0062, p < .01				
d = 1.00				
**lme analysis**				
**Random effects**				
	**Groups Name**	**Variance**	**VPC**	**Corr**

	participant (Intercept)	0.059202	0.349	
	prime/participant	0.001526	0.009	–1.00
	item (Intercept)	0.004365	0.026	
	prime/item	0.000000	0.000	
	Residual	0.104580	0.616	
Number of obs: 4512, groups: ITEM, 120; SUBJECT, 40				
**Fixed effects**				
		**Estimate**	**Std. Error**	**t value**

	(Intercept)	–1.75656	0.03953	–44.43
	REPETITION	0.11161	0.01145	9.75
**Correlation of Fixed Effects**				
		**(Intr)**		
	
	REPETITION	–0.627		
Estimated RTs are: related= –1000/–1.757 = 569ms, unrelated = –1000/(–1.756+.112) = 608ms				
Effect size Westfall et al.: d = \,\,\,\frac{{.112}}{{\sqrt {.05920 + .00153 + .00436 + .00000 + .10458} }}\,\,\, = .272				

A comparison of the VPCs in Adelman et al. (2014) and Perea et al. (2015) indicates that both datasets are very similar. There is some more residual noise in Perea et al. and the VPCs of the slopes of the priming effect across participants and primes are a bit lower than in Adelman et al. This is compensated by some more variance in the intercepts. The order of the VPCs in both datasets is the same: Residual, participant intercept, item intercept, participant slope, and item slope.

An analysis according to Westfall et al. (2014) indicates that the power of the Perea et al. study verges towards 1.00 and that the 39 ms effect could have been found with 9 participants or 13 stimuli. At the same time, the program indicates that the design would lack the power to find a priming effect of one third (d = .09, more or less similar to a priming effect of 13 ms). Then, power reduces to .60.

We can also use simulations to estimate the power of Perea et al. (2015) for smaller numbers of participants and/or items. An interesting command in the simr package for this is powerCurve. The commands below show how to use it:

library(simr)
perea$invRT <-- 1000/perea$RT
fit <- lmer(invRT ~ REPETITION + (1|ITEM) + (REPETITION|SUBJECT), data = perea)
pc1 <- powerCurve(fit, along = “ITEM”, nsim = 50)
plot(pc1)

**Figure 5:**
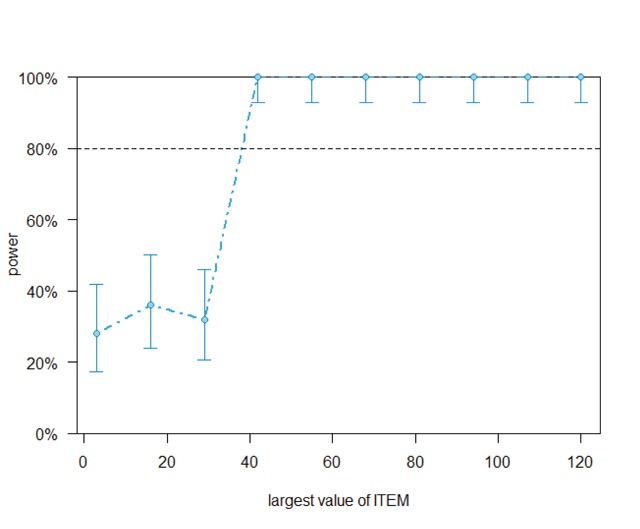
Outcome of the powerCurve command from the simr package for the Perea et al. (2015) dataset. It shows how the power based on the 40 participants tested increases as a function of the number of items. With 40 items we have enough power to observe the 39 ms repetition priming effect.

**Figure 6:**
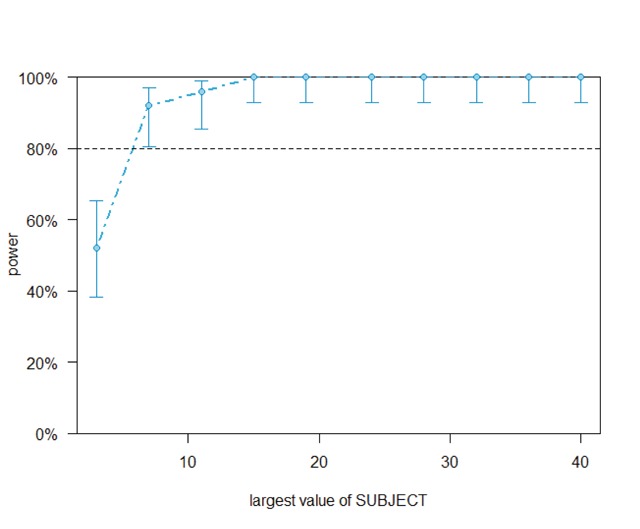
Outcome of the powerCurve command (simr package) for the Perea et al. (2015) dataset. It shows how the power based on the 120 items tested increases as a function of the number of participants. With 7 participants we have enough power to observe the 39 ms repetition priming effect.

Figures 5 and 6 show the outcome. If identity priming had been the only factor Perea et al. wanted to investigate, 40 stimuli would have sufficed. Alternatively, with 120 stimuli 7 participants would have sufficed.

Perea et al.’s (2015) study is overpowered because of the large priming effect. So, an interesting possibility is to see how much power it has for smaller priming effects. The latter can be estimated by adding a constant to the RTs of the related condition, so that the priming effect becomes smaller.^5^ Table 7 shows the outcome. It also shows the power of the Adelman et al. database, when the observations are limited to random samples of 40 participants and 120 stimuli. The analysis shows that both databases behave similarly, but that the power for the Perea et al. database increases more rapidly as a function of the effect size than that of the Adelman et al. database. Keep in mind that the Adelman et al. estimates are based on 100 random samples of 40 participants and 120 stimuli each.

**Table 7:** Power of the Perea et al. study to observe priming effects of various magnitudes. The same information is given for the Adelman et al. study when the number of participants is limited to 40 and the number of items to 120.

	Perea et al.	Adelman et al. (40p:120i)

Priming effect = 5 ms	.25	.32
Priming effect = 7 ms	.44	.45
Priming effect = 10 ms	.80	.61
Priming effect = 12 ms	.89	.81
Priming effect = 15 ms	.91	.90

## Increasing the number of conditions

Psychologists rarely run experiments with two conditions (see Table 8 below). They have a tendency to add extra levels to a variable or to add extra variables. To dip into this realm, we looked at what happens when an extra condition is added to the Adelman et al. dataset. Instead of two levels, we assume the prime variable has three levels: high, medium and low, with the following invRTs: –1.615 (619 ms), –1.596 (627 ms), and –1.562 (640 ms).

**Table 8:** Numbers of participants and trials used in a sample of masked priming studies. Trials limited to those items that were analyzed (e.g., the words in a lexical decision task). The last column shows the number of observations per cell of the design (i.e. per condition tested).

Reference	Study	Task	Nconditions	Npart	Ntrials	Nobs/cell

Bell et al. (2015)	Exp 1	Semantic classification	6	23	120	460
	Exp 2	Semantic classification	6	41	120	820
	Exp 3	Semantic classification	6	39	120	780
Beyersmann et al. (2015)		Lexical decision	8	191	50	1194
Ussishkin et al. (2015)	Exp 2a	Lexical decision	3	66	36	792
	Exp 2b	Lexical decision	3	70	36	840
Kgolo & Eisenbeiss (2015)	Exp 1	Lexical decision	7	70	55	550
	Exp 2	Lexical decision	8	63	55	433
	Exp 3	Lexical decision	8	65	55	446
Sulpizio & Job (2015)	Exp 1	Word naming	6	24	48	192
	Exp 2	Word naming	6	24	48	192
	Exp 3	Word naming	6	24	48	192
	Exp 4	Word naming	9	20	56	124
Dasgupta et al. (2015)		Lexical decision	10	28	490	1372
Guldenpenning et al. (2015)	Exp 1	Action decision	24	44	408	748
	Exp 2	Action decision	48	50	576	600
Atas et al. (2015)		Direction decision	32	29	2560	2320
Perea et al. (2015)		Lexical decision	4	40	120	1200
Kiefer et al. (2015)	Exp 1	Evaluative decision	8	24	384	1152
	Exp 2	Evaluative decision	16	24	768	1152
	Exp 3	Evaluative decision	2	20	150	1500

Potentially there are two advantages of having three levels of a prime rather than two levels. First, the difference in average RT between high and low is larger (21 ms instead of 16 ms). Second, existing power calculators suggest that the power increases when an extra level is added to a repeated measure. For instance, if the effect size is f = .25 (comparable to η^2^ = f^2^ = .0625 or d = .52), the software package G*Power (Faul, Erdfelder, Lang, & Buchner, 2007) advises a sample size of 34 participants when the repeated measure contains two levels (for power = .8). When the repeated measure has three levels, the recommended number of participants drops to 28. To some extent, this is understandable, as adding another observation increases the total number of observations.

In contrast, Bradley and Russell (1998), on the basis of simulations, strongly warned against the introduction of an extra, in-between level to a repeated measure, because it decreases the power of the study.

A second complicating factor is that most researchers increase the number of conditions, but not the number of items (or participants). As a result, the same number of observations is dispersed over more conditions, which in all likelihood will decrease the power. It must not be forgotten that when one condition is added to a software package like G*Power, it is assumed the number of observations increases. This is an another example of where use of software packages designed for designs with one random variable may give misleading information to researchers working with designs that include two random variables.

When we run the simr program on the Adelman et al. database with 40 participants, 78 stimuli and three levels of prime, we get a power estimate of 75% (SE = 2.3) against a power estimate of 80% (SE = 2.4) with two levels (see also Table 5). When we increase the number of stimuli to 117 (so that each condition includes 39 items), power for the three-level design goes to 83% (SE = 1.6).

All in all, our simulations are in line with Bradley and Russell (1998; see also Stevens & Brysbaert, 2016): Adding a third level with an average in-between the other two levels decreases the power of an experiment, particularly when the number of observations remains the same. Therefore we recommend to heed the following cautionary rule: *If the number of conditions in a design is increased, it is better to increase the number of observations to the same extent*. So, when contemplating the required number of observations, we better think of the number of observations per condition rather than per experiment. More complicated designs require more observations.

## Conclusions and recommendations

In this article we described how cognitive psychologists can avoid running underpowered experiments. Such experiments have been too prevalent in the history of psychology and are the main cause of the replication crisis. Arguably, psychology made the wrong choice in the 1990s. Before, it was time intensive to collect and analyze data of experiments. With the advent of computers, everything became much easier and faster. Whereas before it took months to complete a study, now studies could be run in a matter of days (mainly limited by the availability of participants). Instead of running more experiments, however, we should have opted (and required) to run better, more powered experiments. This is the correction we still have to make.

One thing that has become clear from recent analyses is that it is bad practice to use effect sizes of published article as an estimate for power analysis, because they tend to be exaggerated. Much better is to assume effect sizes of d = .4 or d = .3 (the typical effect sizes in psychology), as shown in the introduction. Similarly, in cognitive psychology it is better to start from a typical effect size. For instance, many masked priming studies result in priming effects of 10–20 ms. These are the values to build upon.

On the basis of an analysis of big datasets coming from large-scale studies and our research experience, we have come to the following recommendations related to experiments based on reaction times (RTs):

1. The variance in RTs relative to the effect sizes is considerable. Standard deviations of RTs are typically in the 150–200 ms range, even for simple tasks with mean RTs around 600 ms. Standard deviations are larger for tasks or participants with slower RTs, as there is a positive correlation between the mean and the standard deviation of the RT distribution. This means that standardized effect sizes are commonly around d = .1, which are very small (Cohen defined d = .2 as a small effect size).
2. Such effect sizes can be detected by having enough observations. We recommend a minimum of 1,600 observations per condition in designs with repeated measures. This will allow researchers to interpret differences of some 15 ms. Within limits, the number of observations can be divided over participants and items (20 participants and 80 items, 40 participants and 40 items, or 80 participants and 20 items), depending on how easy it is to get access to one or the other.
3. Mixed effects analyses are more powerful than separate F1 and F2 analyses (Stevens & Brysbaert, 2016). They are not more difficult to run anymore and better fit the data (Judd et al., 2017).
4. In our analyses, we saw that working with invRT as dependent variable increased the power of the analysis. There is currently a debate about whether or not transformations of dependent variables are required or should be avoided. Some authors demand the use of transformations to get normal distributions (e.g., Baayen, 2008). Others warn that transformations may alter the nature of the data (e.g., a logarithmic transformation may alter the interactions between independent variables; Lo & Andrews, 2015). Another way may be to work with mixed models that take into account non-normal distributions (Lo & Andrews, 2015). What must be avoided, is that authors try out the various options and only report the one that produces a “significant” effect, a phenomenon known as p-hacking (Simmons et al., 2011). Our standard approach is only to interpret data that are significant both in the transformed and the untransformed analysis (unless there are clear theoretical reasons for one or the other). A consequence of this approach is that we would rather opt for the numbers of observations provided in Table 4 than those in Table 5 (i.e. 3,000 observations per condition rather than 1,600 observations per condition).
5. When working with RTs it looks like Westfall et al. (2014) give too optimistic estimates, possibly because the contributions of the variances in intercepts and slopes is much smaller than the values social psychologists are used to. Better to work with simulation. Easy-to-use packages have become available to do so. The data can come from simulations (as in Stevens & Brysbaert, 2016) or from the datasets provided in the present article.
6. Simulations work best when you have pilot data to work with. This tailors the analysis to the problem you are investigating. We have shown how you can use the output of an lmer analysis to calculate the power of your design. We think it would be good practice if researchers always published the full outcome of their lmer analysis in their articles (as shown in Tables 2, 5, and 6). In this way, we can build a better understanding of the VPCs obtained in typical paradigms. For instance, we have observed that the two datasets related to masked orthographic priming returned comparable VPCs. These are a good basis for the interpretation of new datasets. It would be good if we had similar estimates for other paradigms, both in psycholinguistics and in other areas of cognitive psychology.
7. Effect sizes in existing meta-analyses are overestimated because they are mainly based on F1 analyses, the estimates of which depend on the number of stimuli used in the various conditions. For instance, Wen and van Heuven (2017) reported an average effect size of d = .86 for masked translation priming from words in the first language to words in the second language. Our analysis in Table 3 indicates that this estimate is dependent on the number of stimuli presented to the participants (as, indeed, reported by Wen & van Heuven, 2017). So, the estimate can only be used for a power analysis if you intend to present the same number of stimuli. Similarly, if you use an effect size based on F2 analyses for your power evaluation, you must test the same number of participants as in the original study/studies. The simulations of Table 3 also show that the heterogeneity of the effects in a meta-analysis will be overestimated, if differences in stimulus and participant samples are not taken into account.

To see how far our recommended number of observations (minimally 1,600 per condition) deviates from current practice, we checked 10 articles on masked priming in the Web of Science (search for the most recent papers done on December 15, 2015). Table 8 lists the results. As can be seen, only one study met the requirement, even though many studies were investigating small effects. Indeed, much research in cognitive psychology concerns searching for the boundary conditions of more robust effects (which by definition involve small effect sizes). A similar conclusion was recently reached by Mahowald, James, Futrell, and Gibson (2016) for syntactic priming.

From our experience, we know that psychologists will be very creative in finding reasons why the number of observations can be lower in their research. Unless these are based on a proper power analysis, it is to be expected that many of these “reasons” will be fallacies aimed at decreasing the work load rather than cherishing the quality of the research done (Smaldino & McElreath, 2016). Indeed, the history of psychology’s accomplishments thus far should remind us that our tendency to run underpowered studies is a much bigger problem than the risk of some resource waste due to a needlessly overpowered study (keeping in mind that a power of 80% still entails a risk of 20% that the true effect will not be found).

It is also good to keep in mind that the recommendations we give are limited to repeated measures designs in which the stimuli are presented in all conditions, such as in priming studies. If a variable is between stimuli (e.g., in a study on high-frequency vs. low-frequency words), more observations are needed. Some estimates for this situation were given by Keuleers, Lacey, Rastle, and Brysbaert (2012, see also Keuleers, Diependaele, & Brysbaert, 2010). They ran a power analysis on the British Lexicon Project, which consists of a lexical decision experiment, in which 40 participants responded to 14 thousand words and 14 thousand nonwords each. Keuleers et al. (2012) observed that with a group of 40 participants, one needs 160 words and 160 nonwords per condition to find a word frequency effect of 20 ms with a power of .80 (this is a total of 40 * 160 * 4 = 25,600 observations, of which half are not used in the analysis, namely the data of the nonwords). With 40 participants and 40 words per condition, the frequency effect had to be 40 ms before it could be detected reliably. This is another reminder that a shortage of observations is a much more serious problem in experimental psychology than an excess of observations.

## Additional Files

The Additional files for this article can be found as follows:

10.5334/joc.10.s1 Adelman with two levels. An Excel file containing all observations of the Adelman et al. (2014) study with the prime conditions simplified to high and low.

10.5334/joc.10.s2 Adelman with three levels. Another Excel file containing the observations of Adelman et al. (2014) with three levels of priming: high, medium, low.

10.5334/joc.10.s3 Analysis Adelman. An R file to analyze the two previous Excel files (can be opened with all text processors).

10.5334/joc.10.s4 Perea data. Excel file containing all observations of the Perea et al. (2015) study.

10.5334/joc.10.s5 Analysis Perea. An R file to analyze the Perea et al. data.

The files are also available at: https://osf.io/fhrc6/. They can be used to start simulations aimed at the specific questions individual researchers may have or to check the correctness of analyses done on other datasets.

## Footnotes

For comparison, when you compare random groups of 4 men and 4 women, you have 80% chance of observing a p < .05 significant difference in their heights.

The authors thank Jason Geller for pointing them to this package.

Simulations, of course, can be applied to the full design, for instance to check whether the study was powerful enough to observe an interaction under the assumption that the priming effect with alternating case primes was half the size of that with lowercase primes. Some examples of the power for interaction terms are given in Stevens & Brysbaert (2016).

If this is not done, the model often fails to converge and then all variability is given to the slope per item instead of to the intercept.

Notice that this technique can also be used to find the power of observing the interaction effect Perea et al. (2015) were looking for. It then suffices to add, for instance, 19.5 ms to half of the stimuli preceded by a related prime. This allows the researcher to estimate the power of the experiment for finding the interaction in the assumption that the repetition priming effect halves for case alternated primes.
